# Management of Turner's Hypoplasia Using Resin Infiltration: A Case Report

**DOI:** 10.7759/cureus.48870

**Published:** 2023-11-15

**Authors:** Dhruvi Solanki, Punit Fulzele, Nilima Thosar, Unnati Shirbhate

**Affiliations:** 1 Department of Pediatric and Preventive Dentistry, Sharad Pawar Dental College, Datta Meghe Institute of Higher Education and Research, Wardha, IND; 2 Department of Periodontics, Sharad Pawar Dental College, Datta Meghe Institute of Higher Education and Research, Wardha, IND

**Keywords:** enamel hypoplasia, intrinsic tooth stains, esthetic dentistry, anterior discoloration, turner’s hypoplasia, turner’s tooth, white spot lesion, resin infiltration technique

## Abstract

The causes of enamel discoloration can vary, leading to aesthetic concerns for patients. Injuries to primary teeth can lead to developmental issues in permanent successors, with enamel hypoplasia, commonly referred to as Turner's tooth. Diverse methods are available for addressing tooth discoloration. A case of an 11-year-old pediatric patient with a brown patch on the upper left central incisor was reported to the Pediatric Dentistry Department. A well-demarcated, yellowish-brown lesion was present on the labial surface of 21 and was diagnosed as Turner’s hypoplasia. Resin infiltration was done using the Icon Smooth Surface (DMG America Company, Englewood, NJ) resin infiltration kit. The resin infiltration technique provides an approach to meet aesthetic requirements. In this case, the resin infiltration technique was successfully utilized to address the discoloration of the left maxillary central incisor, which was affected by Turner's hypoplasia.

## Introduction

Dental practitioners often encounter cases of enamel staining and discoloration in children. The causes of enamel discoloration can vary, leading to aesthetic concerns for patients. External sources like coffee, tea, and tobacco can stain enamel. At the same time, intrinsic factors, whether congenital, like amelogenesis imperfecta, or acquired, such as tetracycline staining or primary tooth pathology, can also be responsible. Historically, these color irregularities were addressed through restorative materials, providing satisfactory aesthetics. However, these restorations necessitated frequent repairs and replacements, perpetuating the cycle of dental restoration [1]. Single-tooth discoloration can stem from various conditions, such as intrapulpal bleeding after injury, leading to the deposit of degraded blood products like hemosiderin, hematin, and hemine; pulp death resulting in chromogenic byproducts; remnants of cement or gutta-percha in the pulp chamber; incomplete removal of pulp; formation of reddish-brown precipitate due to sodium hypochlorite and chlorhexidine combination; caries; faulty restorations; cervical resorption; calcific metamorphosis; enamel defects; and misaligned teeth, prone to external staining and shadowing [2,3].

There are diverse methods available for addressing the discoloration of a single tooth. Treatment choice depends on several factors, such as the underlying source of discoloration, the dentist's experience, the patient's budget and interest, the amount of natural tooth structure still present, and past dental procedures. Given the rising preference among patients for minimally invasive solutions, prioritizing therapies with little biological impact is essential [3,4].

## Case presentation

**Figure 1:**
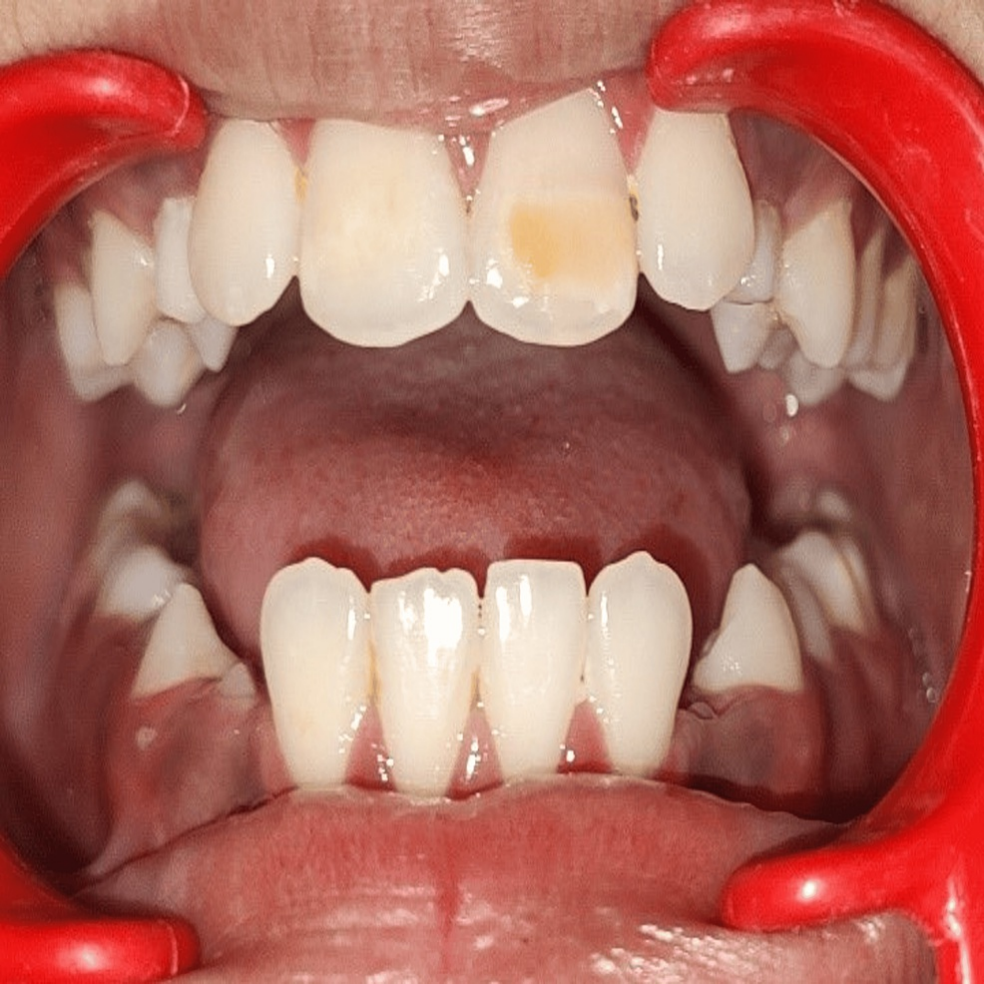
Intraoral image showing Turner's hypoplasia in relation to the maxillary left central incisor.

**Figure 2:**
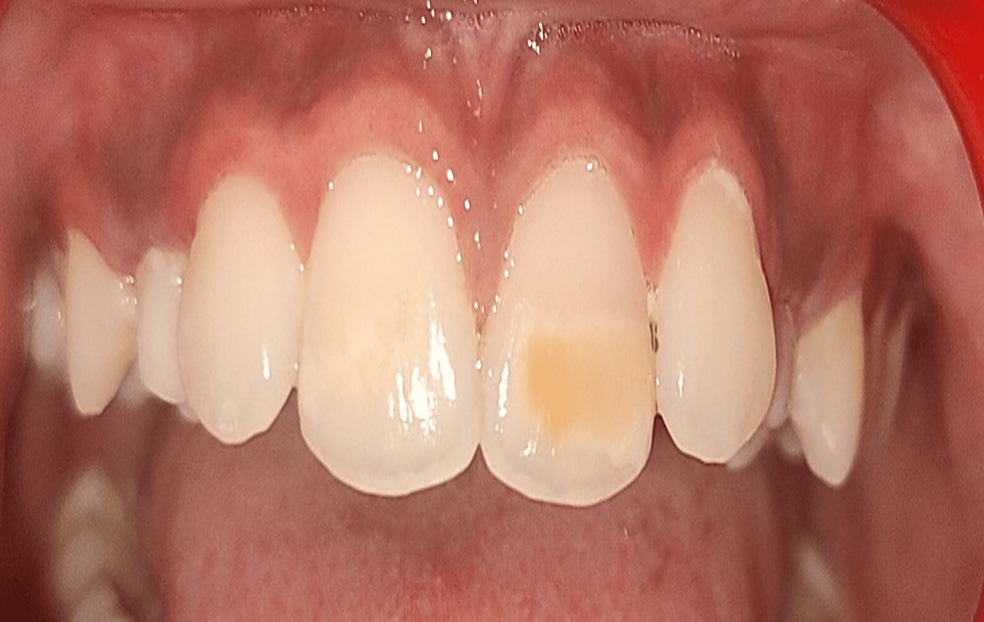
Yellowish-brown discoloration in relation to the upper left central incisor.

An 11-year-old male patient presented with a concern about a brown patch on his upper front tooth that had persisted for four years, undermining his confidence. The patient had class III malocclusion. Intraoral clinical examination of the patient revealed mixed dentition, which was caries-free, and teeth numbers 43, 44, and 45 were erupting. A well-demarcated, yellowish-brown lesion was present on the labial surface of tooth number 21, involving the middle and incisal-third of the tooth with an incipient caries lesion on the distal aspect. During the history-taking process, the parents disclosed that the child had fallen on his mouth two years ago, as illustrated in Figure 1 and Figure 2. 

Upon vitality testing, the tooth responded normally. Based on the gathered data, a diagnosis of Turner’s hypoplasia for tooth number 21 was provided. The patient, along with his parents, was briefed on various treatment options such as bleaching, micro-abrasion, resin infiltration, and composite. They ultimately chose resin infiltration as the preferred treatment. 

**Figure 3:**
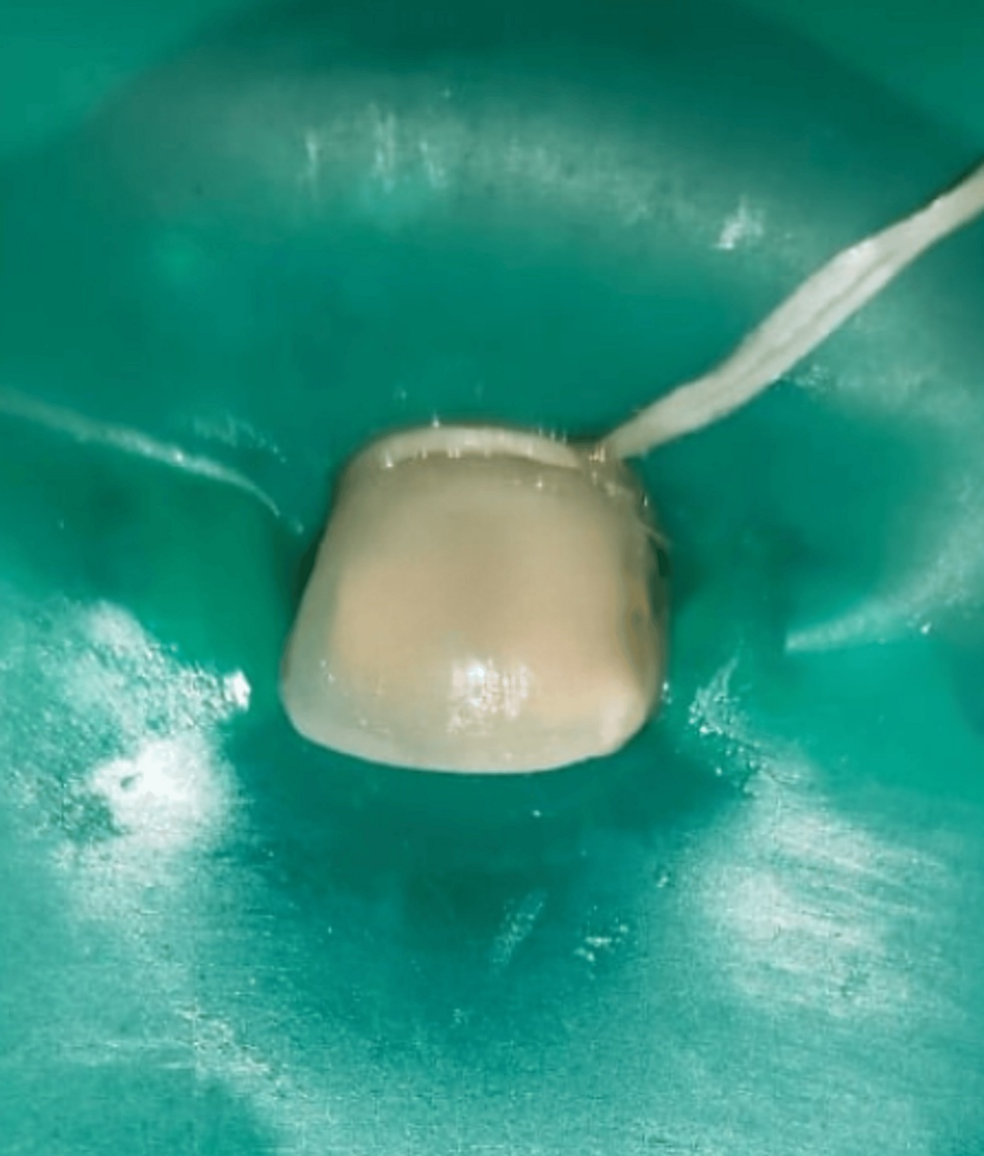
Intraoperative image of the upper left central incisor after rubber dam isolation and resin infiltration.

The treatment began with preventive procedures, including complete oral prophylaxis and the application of pit and fissure sealant on all permanent molars. Proper rubber dam isolation was carried out to prevent saliva interference, and instrument swallowing was addressed. Following this, Icon-Etch (Icon Smooth Surface, DMG America Company, Englewood, NJ) was applied to the dried surface of the lesion for two minutes. The etchant was then suctioned, and the tooth was washed and dried with air. Next, Icon-Dry was administered, left in place for 30 seconds, and then dried. Subsequently, the tooth was treated with Icon-Infiltrant and left on for five minutes. Two applications of infiltration were completed within these five minutes to ensure sufficient incorporation of the material into the lesion. Any excess material was removed, and then the area was exposed to light curing for 40 seconds, as illustrated in Figure 3.

**Figure 4:**
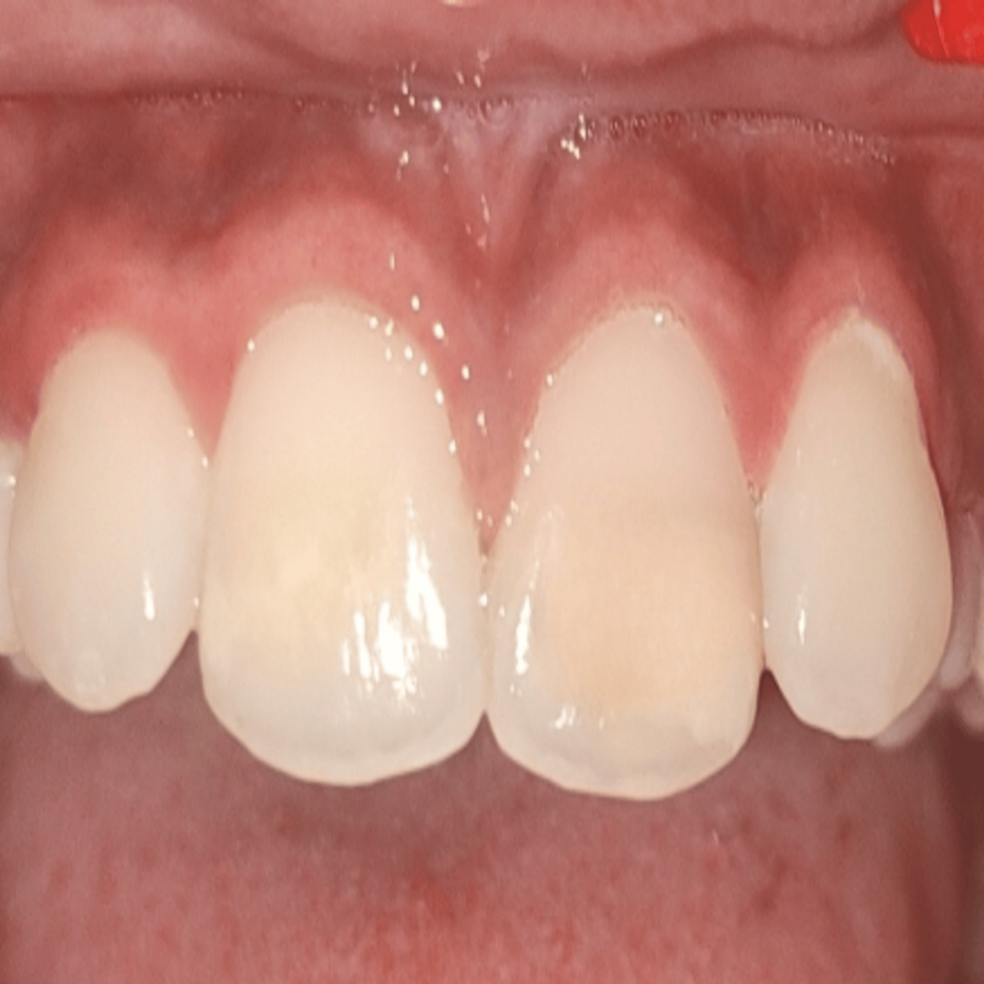
Postoperative image of the upper left central incisor after finishing and polishing.

This was followed by finishing and polishing. Postoperatively, tooth number 21 looked esthetically similar to 11, as shown in Figure 4. The patient was instructed to abstain from colored foods and beverages for five days.

## Discussion

Injuries to primary teeth can lead to developmental issues in permanent successors, with enamel hypoplasia, commonly referred to as Turner's tooth, being the most frequent malformation. In Turner's hypoplasia, the enamel may appear pitted, rough, or glossy and can affect either a portion or the entire crown of the tooth. This condition is frequently observed in maxillary incisors as well as molars. Obtaining a comprehensive history, particularly concerning the discolored tooth, is crucial for diagnosing the cause of discoloration accurately [5].

Ogaard et al. cautioned against using concentrated fluoride agents to treat white lesions on labial surfaces. Such treatment might halt the lesion's progress (hypermineralization) and impede complete repair, particularly for deep lesions, which tend to only superficially remineralize [6]. Enamel microabrasion was developed to enhance surface texture, eliminate stains, and facilitate remineralization. It involves removing superficial parts of the lesion through abrasion using a mixture of hydrochloric acid and pumice, resulting in a smooth, glossy enamel surface. This process uniformly removes up to 0.2 mm of the enamel surface [2].

The resin infiltration technique provides an alternative approach to meet aesthetic requirements. This method aims to establish a diffusion barrier inside the lesion rather than just on its surface. Commercial products like ICON are available for this purpose, effectively reducing the visibility of these lesions. This technique employs newly developed light-polymerized resins to fill the micropores created by an acid. It is suitable for treating *spots* on smooth enamel surfaces but is not recommended for cavitated enamel or deep-seated lesions. ICON consists of three constituents: Icon-Etch comprising hydrochloric acid, pyrogenic silicic acid, and a surface-active substance; Icon-Dry, predominantly composed of 99% ethanol; and Icon-Infiltrant, which contains a resin matrix based on methacrylate and an initiator [7,8].

Special care is required while working with Icon-Etch, as it can lead to pain and discomfort to the patient if applied on dentin. Additionally, it's crucial to ensure that the lesion is thoroughly cleaned and isolated because the presence of organic materials like proteins and carbohydrates can hinder resin penetration. This technique was initially developed and implemented by Kielbassa et al. and Paris et al. [9,10]. White spot lesions, hypomineralization, and hypoplastic defects appear opaque due to the difference in refractive indices between enamel crystals and the inside of the pores within these lesions. Usually, water or air is contained within these micropores. The resin infiltration method involves infusing these micropores with a low-viscosity resin. Consequently, the refractive index difference between the micropores and the enamel is abolished, and the lesion appears similar to natural enamel for a longer period of maintenance [2,8].

Unfortunately, neither microabrasion nor resin infiltration techniques can completely eliminate white spot lesions. This residual presence post-treatment might be due to the white spot lesions not being confined to the enamel's superficial layer. Studies indicate that microabrasion removes only 200 μm of the enamel's surface, and resin infiltration reaches a depth of approximately 60 μm. If a white spot lesion extends beyond these depths, it might still be visible. Hence, careful consideration in selecting cases is necessary [11,12].

## Conclusions

In this case, the resin infiltration technique was successfully utilized to address the discoloration of the left maxillary central incisor, which was affected by Turner's hypoplasia. Applying this technique restored the natural appearance of the affected tooth and underscored the effectiveness of this approach in managing discolorations. This successful case adds to the body of evidence supporting the efficacy of resin infiltration in masking enamel discolorations. It highlights its potential as a reliable solution for similar cases in dental practice. It offers a less invasive option that can be performed at an earlier stage in tooth growth compared to dental veneers, which require waiting until tooth growth is complete.
